# Re-emerging and newly recognized sexually transmitted infections: Can prior experiences shed light on future identification and control?

**DOI:** 10.1371/journal.pmed.1002474

**Published:** 2017-12-27

**Authors:** Kyle Bernstein, Virginia B. Bowen, Caron R. Kim, Michel J. Counotte, Robert D. Kirkcaldy, Edna Kara, Gail Bolan, Nicola Low, Nathalie Broutet

**Affiliations:** 1 Division of STD Prevention, Centers for Disease Control and Prevention, Atlanta, Georgia, United States of America; 2 Department of Reproductive Health and Research, World Health Organization, Geneva, Switzerland; 3 Institute of Social and Preventive Medicine, University of Bern, Bern, Switzerland

## Abstract

How do we spot the next sexually transmitted infection? Kyle Bernstein and colleagues look for lessons from past discovery.

**Summary points**

- Determining sexual contact as a mode of pathogen transmission and quantifying the risk of sexual transmission pose epidemiologic challenges.
- Prior experiences with nontraditional sexually transmitted infections present valuable epidemiologic lessons, including comparisons of disease rates by sex, molecular analyses among sexually linked clusters, and methods to control for other potential modes of transmission.
- Applying lessons learned from prior infections might be critical for rapid and effective detection, prevention, and control of other reemerging and newly recognized sexually transmitted infections.

The spectrum of pathogens that have a sexually transmitted component is broad. Globally, there are more than 30 recognized sexually transmitted infections (STIs), including those transmitted primarily by sexual contact and those that are sexually transmissible but whose primary mode of transmission is by food, vector, or droplet [1]. This latter group of nontraditional STIs poses unique methodologic and epidemiologic challenges for public health practitioners and researchers, who need to anticipate, identify, and contain the next new STI outbreak. In this paper, we explore these challenges using examples of nontraditional STIs, including 2 (shigellosis and *Neisseria meningitidis*) that have recently reemerged as sexually transmissible and 2 (Zika and Ebola) that are newly recognized as being sexually transmissible (Table 1).

**Table 1:** Modes of emergence and key characteristics of selected sexually transmitted infections.

	HIV	*N*. *meningitidis*	*Mycoplasma genitalium*	*Shigella* spp., *Entamoeba histolytica*, and *Giardia lamblia*	Zika virus	Ebola virus
Mode of emergence	New pathogen in humans, newly recognized disease	Newly discovered pathogen, known genital disease	Newly discovered pathogen, known genital disease	Recognized pathogen, extragenital site	Recognized pathogen, newly recognized mode of transmission	Recognized pathogen, newly recognized mode of transmission
Mode of discovery	New disease, AIDS, United States, 1981 [2]. HIV transmission began much earlier in sub-Saharan Africa but was unrecognized [3].	Isolation from men with urethritis, US, 1942 [4]	Isolation from men with nongonococcal urethritis, United Kingdom, 1981 [5]	Outbreaks in MSM, proctitis, US, 1960s and 1970s [6–9]	Sexual partner in US of returning traveler from Senegal, 2011 [10]	Tail end of epidemic, Liberia, 2015 [11]
Year pathogen first reported	1983 [11]	1942 [4]	1981 [5]	Early 20th century	1947 [12]	1977 [13]
Reservoir	Human	Human	Human	Environmental	Human	Zoonotic
Primary portal of entry/exit	Mucosal surfaces (genitourinary and anorectum); percutaneous	Mucosal surfaces (mouth and genitourinary)	Mucosal surfaces (genitourinary and anorectum)	Mucosal surfaces (mouth and anorectum)	Percutaneous	Mucosal surfaces
Primary mode of transmission	Direct contact (sexual intercourse), injection, and vertical transmission	Direct contact (sexual intercourse) and droplet transmission	Direct contact (sexual intercourse)	Ingestion	Vector-borne (mosquito)	Direct contact (touching)
Disease	HIV infection and AIDS	Nonspecific urethritis and invasive meningococcal disease	Nonspecific urethritis	Proctitis	Zika virus infection	Ebola virus disease
Type of disease	Systemic	Genital and systemic	Genital	Extragenital	Systemic	Systemic
Genital symptoms	No	Yes, when the infection is localized to the genital tract; no genital symptoms in the systemic (invasive) form of the disease	Yes, typical	Extragenital	Not usual; hematospermia reported	Not usual
Persistence in genital secretions	Lifelong	Not known	Not known; up to a year	Not present in genital secretions	Usually less than a year	Usually less than a year
Curable?	No	Yes	Yes	Yes	No	No

MSM, men who have sex with men.

## *Shigella*: Raised male-to-female ratios in routine surveillance data

Shigellosis is a diarrheal illness caused by several species of the bacterium *Shigella*. *Shigella* is transmitted by direct or indirect contact with human feces, often via contaminated food, water, or fomites [13]. Prior to the 1970s, *Shigella* incidence was highest among children <5 years of age, their caretakers, and travelers to less developed countries. Recognition of *Shigella* as a potential STI began in the 1970s with outbreaks among men who have sex with men (MSM) in the US [8, 9, 14, 15]. Sexual transmission of *Shigella* likely occurs during oral-anal sex (e.g., anilingus or rimming) or digital-anal sex (e.g., fisting) [16, 17]. During the 1970s and 1980s, *Shigella flexneri* rates increased in the US among adult males, even as overall rates and rates among children declined [18]. Routine case reports for *Shigella* do not include information about sexual practices, but the widening disparity between adult male and female case rates strongly suggested male-male sexual transmission. Increases in *Shigella* among men in the US and England between 2004 and 2015—despite declining or steady rates among women and children—support the reemergence of *Shigella* as an STI among MSM [19, 20]. *Shigella* strains among MSM have demonstrated increasing multidrug resistance over the past 5 years [21–28], and recent genomic analyses suggest international spread of an antimicrobial-resistant *S*. *flexneri* serotype among MSM [23].

## *N*. *meningitidis*: The role of dyad and cluster analyses

*N*. *meningitidis*, the bacterium that causes invasive meningococcal disease (IMD), spreads primarily by droplet transmission and infection of respiratory mucosa. Approximately 5%–10% of healthy adults are nasopharyngeal carriers. *N*. *meningitidis* has been isolated from men with urethritis [4]. A 1972 study described the transmission of *N*. *meningitidis* from a male chimpanzee’s nasopharynx to his own urethra via oral-genital autoinoculation [29]. The authors concluded that *N*. *meningitidis* in the human urogenital tract might be the result of oral-genital sexual contact. Dyad and cluster analyses showing related strains of meningococci among partners epidemiologically linked by female-to-male oral sex strengthen the argument for sexual transmission leading to meningococcal urethritis [30, 31]. Recent molecular analyses suggest that *N*. *meningitidis* has genetically adapted to the urogenital tract [32]. Recent IMD outbreaks among MSM in Europe, Canada, and the US have also raised questions about the role that sexual networks play in *N*. *meningitidis* transmission [33–38]. Droplet transmission within MSM sexual networks could explain consistently higher *N*. *meningitidis* nasopharyngeal carriage rates relative to heterosexual men [39, 40]. It remains challenging, however, to determine whether the primary mode of transmission in IMD outbreaks among MSM is oral-genital contact, open-mouth kissing, or droplet transmission via “close contact,” including the sharing of living and sleeping spaces.

## Ebola virus: Viral persistence in semen and genetic epidemiology

The largest outbreak of Ebola virus disease started in December 2013 in West Africa and led to >28,000 confirmed cases and 11,310 deaths [41]. Almost all infections resulted from exposure to acutely symptomatic infected persons or recently deceased Ebola patients. Concern about possible sexual transmission of Ebola grew as the outbreak continued [42]. Anecdotal reports of new Ebola infections occurring among persons not in close proximity to a symptomatic or recently deceased person were followed by a report of a Liberian woman with Ebola, whose only possible source of infection was her husband, a convalescing Ebola survivor [43]. The husband had a positive PCR test for Ebola RNA in his semen 199 days after symptom onset, and the homology between genetic sequences of the Ebola RNA from the man and woman suggested that the only possible source of her infection was through sexual transmission [43, 44]. Ebola virus persistence in the semen of male survivors was documented in previous sporadic outbreaks [45, 46]. However, in the most recent West African outbreak, more robust systematic assessments found male survivors with Ebola virus RNA detected by PCR up to 565 days after symptom onset [47–49]. There is little evidence supporting viral persistence in other body fluids [42]. Female-to-male sexual transmission of Ebola is likely inefficient, but data are limited. Although the risk of transmission from semen exposure is considered small, the sheer number of male Ebola survivors raised concern about potential flare-ups and new clusters as the West African outbreak waned [50, 51]. Little is known about the public health impact of Ebola persistence among high-risk groups such as sex workers and MSM.

## Zika virus: Infections in sexual partners of travelers returning from endemic areas

A large outbreak of Zika virus in Latin America and the Caribbean in 2015–2016 drew international attention because of its reported association with microcephaly. By 2017, 84 countries and territories had evidence of Zika transmission [52]. While the predominant mode of Zika transmission is through the bite of an infected *Aedes* spp. mosquito, sexual transmission was documented when a scientist returned to the US from Senegal in 2008 and transmitted Zika to his female sex partner who had not travelled [10]. Case reports from 13 countries have since described probable sexual transmission of Zika—via oral, anal, and vaginal sex—to partners of travelers returning from endemic areas. Suspected male-to-female sexual transmission was reported in 27 couples, while only 1 case of female-to-male and 1 case of male-to-male sexual transmission have been documented [53]. Most sexual transmission events occurred in symptomatic couples, but the timing of suspected transmission relative to symptom onset ranges greatly.

Persistent detection of Zika in genital fluids by reverse transcriptase (RT)-PCR and culture provides additional evidence for the biological plausibility of sexual transmission. In Puerto Rico, Zika was detected in the semen of 56% of convalescing men, with a median of 34 days between symptom onset and undetectable virus levels [54]. The maximum reported durations of RNA detection in genital fluids are as follows: 188 days in semen by RT-PCR [55], 69 days in semen by culture [56], 3 days in vaginal fluid in RT-PCR, and 11 days in cervical mucus by RT-PCR [57]. Investigation of Zika sexual transmission is complicated by several factors, including difficulty distinguishing vector and sexual transmission in endemic settings and difficulty obtaining viral cultures to confirm that viral persistence represents infectiousness. It is unclear whether controlling sexual transmission of Zika will contribute substantially to overall control of Zika epidemics; mathematical models estimate the population attributable risk of sexual transmission to be from 3% to 23% [58, 59]. Sustained sexual transmission of Zika is unlikely in a general population, but clusters may occur within high-risk sexual networks [60].

## Lessons learned to inform future efforts

Lessons learned from the STIs discussed above can help the public health community identify newly emerging STIs and estimate the potential for an epidemic by sexual transmission. Sexual transmissibility of Zika and Ebola viruses was identified when infections were detected in persons who could only have been infected through sexual intercourse. In the case of meningococcal urethritis, detection of the pathogen in an unexpected anatomic niche and molecular linkage between index patients and their sexual partners established sexual transmissibility. Sexual transmission of *Shigella* was identified by attention to epidemiological changes in the affected populations. *M*. *genitalium*, another predominantly sexually transmitted pathogen, was discovered in 1981 by scientists searching for causes of nonspecific urethritis (Table 1) [56]. This discussion harkens back to the global HIV/AIDS epidemic. HIV crossed a species barrier and spread undetected as a new pathogen and STI among humans for years, before its identification as AIDS in MSM with unexplained immune suppression in San Francisco in 1981 [2,3].

Now is the optimal time to establish methods to assess whether an infectious agent is sexually transmissible and prepare for a new STI with pandemic potential. New or reemerging pathogens that are sexually transmissible will continue to arise. Since HIV was discovered, new molecular tools, such as phylogenetics and the omics, have become available to complement etiological epidemiological investigations of causal associations. For example, genotypic data were critical in linking sexual partners in the context of Ebola [44] and *Shigella* [23]. Standardized definitions and approaches to the investigation of sexual transmission of infectious agents and criteria for considering a pathogen to be sexually transmissible would greatly aid this effort. For example, is transmission by close, intimate contact (i.e., skin-to-skin) considered sexual transmission? Bradford Hill’s classic viewpoints about causation could be adapted; for example, molecular concordance of strains between sexual partners might be a requirement for demonstrating specificity of association. The establishment of an objective criteria for determining the necessary components to consider a pathogen sexually transmitted could complement the existing framework developed by Hill. If sexual transmission is established, quantitative information about parameters such as incubation period, serial interval, transmission probability per coital act, and reproductive number will be needed for mathematical modelling studies of transmission dynamics, the proportion of cases attributable to sexual transmission, the potential for epidemic spread, and the effects of control measures. These quantities are particularly difficult to estimate when the agent has an alternative, predominant mode of transmission in endemic areas, e.g., mosquito-borne Zika. Developing criteria and methodologic approaches to sexual transmission could prove invaluable if they can be applied to key pathogens with epidemic potential, including those that the World Health Organization has published in its blueprint of action to prevent epidemics [61]. A proactive initiative to understand the potential for sexual transmission of such pathogens will help us to stay ahead of the curve.

## Abbreviations

IMD: invasive meningococcal disease
MSM: men who have sex with men
RT: reverse transcriptase
STI: sexually transmitted infection
